# Photo-Crosslinkable Hydrogels for 3D Bioprinting in the Repair of Osteochondral Defects: A Review of Present Applications and Future Perspectives

**DOI:** 10.3390/mi13071038

**Published:** 2022-06-29

**Authors:** Gang Tan, Jing Xu, Qin Yu, Jieyu Zhang, Xuefeng Hu, Chenwei Sun, Hui Zhang

**Affiliations:** 1 Department of Orthopedic Surgery and Orthopedic Research Institute, West China Hospital and West China School of Medicine, Sichuan University, Chengdu 610041, China; tg-828@126.com (G.T.); huaxi4kang@126.com (Q.Y.); 2 Department of Orthopedics, West China School of Public Health and West China Fourth Hospital, Sichuan University, Chengdu 610041, China; 3 Operating Room, West China Hospital, Sichuan University, Chengdu 610041, China; 18502875660@163.com; 4 National Engineering Research Center for Biomaterials, Biomaterials Building, Sichuan University, 29 Wangjiang Road, Chengdu 610064, China; hxsssajj@126.com (J.Z.); huaxi4yiyuan@126.com (X.H.); 5 College of Biomedical Engineering, Sichuan University, 29 Wangjiang Road, Chengdu 610064, China

**Keywords:** photo-crosslinkable hydrogel, 3D bioprinting, osteochondral defects

## Abstract

An osteochondral defect is a common and frequent disease in orthopedics and treatment effects are not good, which can be harmful to patients. Hydrogels have been applied in the repair of cartilage defects. Many studies have reported that hydrogels can effectively repair osteochondral defects through loaded cells or non-loaded cells. As a new type of hydrogel, photo-crosslinked hydrogel has been widely applied in more and more fields. Meanwhile, 3D bioprinting serves as an attractive platform to fabricate customized tissue-engineered substitutes from biomaterials and cells for the repair or replacement of injured tissues and organs. Although photo-crosslinkable hydrogel-based 3D bioprinting has some advantages for repairing bone cartilage defects, it also has some disadvantages. Our aim of this paper is to review the current status and prospect of photo-crosslinkable hydrogel-based 3D bioprinting for repairing osteochondral defects.

## 1. Introduction

Articular cartilage (AC) is an elastic tissue that consists of spheroid chondrocyte cells (2% of the total volume of the AC) protected by the surrounding extracellular matrix (ECM) [1]. Its main function is to transmit loads to the related subchondral bone and absorb impact forces, resulting in low-friction gliding between the surfaces of the joints. AC is characterized by a limited intrinsic regenerative capacity after injury because it is devoid of nerves, blood, and lymphatic vessels [2,3,4,5]. So, large cartilage defects causing cartilage loss remain a therapeutic challenge. 

At present, there are many methods to treat cartilage defects in the clinic. For example, non-surgical approaches include intra-articular injections of various compounds [6], hyaluronic acid (HA) [7], and platelet-rich plasma [8]. The surgical approaches include micro-fracture [9], osteochondral allograft [10] or allografting, matrix-induced autologous chondrocyte implantation [11,12,13,14], and cartilage tissue engineering (CTE) [15].

There are several treatment methods currently available such as lifestyle changes, medication, and osteotomy [16]. However, none of them is successful in recreating native cartilage. Recent research efforts showed that CTE is still one of the most promising fields of research but is also plagued by considerable unsolved challenges that hinder its implementation in clinical practice [17]. Tissue regeneration using advanced scaffolds, growth factors and nutrients, and progenitor or stem cells, provides an alternative treatment option for effectively recreating native cartilage. Hydrogel is the most widely studied and applied material to be scaffolded in the field of cartilage repair and there are two approaches to regenerate cartilage onto scaffolds, namely two-dimensional (2D) and 3D scaffolds [18], and the application of hydrogels coupled with three-dimensional (3D) printing technologies represents a modern concept in scaffold development in CTE. 

3D bioprinting, which not only provides adjustable 3D organizational structures but also encapsulates cells and growth factors, brings forth a new strategy to design biomimetic scaffolds for cartilage repair [19]. Three-dimensional bioprinting can be considered an additive manufacturing technique where biomaterials, cells, and growth factors, often referred to as “bioink”, are printed to create tissue-like structures that imitate natural tissues [20]. 

**Figure 1:**
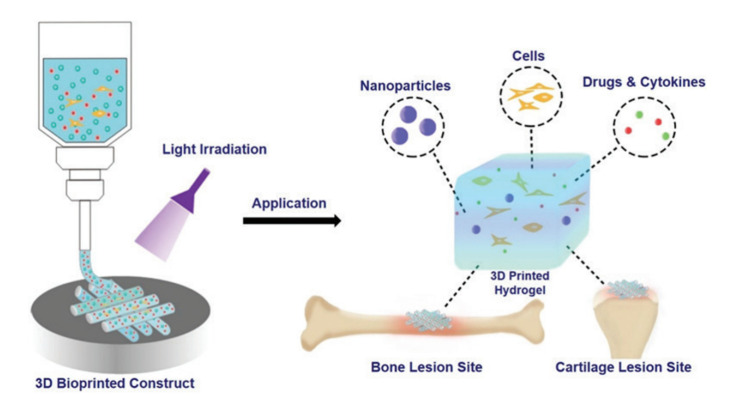
Schematic illustration of photo-crosslinkable hydrogels for the bioprinting of bone and cartilage [23].

Hydrogel is a class of 3D mesh polymer formed by physical or chemical crosslinking that can absorb a large amount of water and maintain its 3D structure [21]. A variety of natural biomaterials and synthetic materials can be used to prepare hydrogels and it is mainly due to their excellent biocompatibility, inherent bioactivity, and special microstructure that they can support tissue regeneration. The use of natural biomaterials represents an attractive strategy for scaffold formation as they mimic the structure of ECM and guide cell growth, proliferation, and phenotype preservation. The photo-crosslinked hydrogel is a kind of hydrogel that triggers a gelation reaction under the action of visible or ultraviolet light and the photoinitiator releases free radicals and combines with the hydrogel prepolymer. Because of its strong adjustability and good biocompatibility [22] and the feasibility of in situ formation and injection delivery, together with its compatibility with existing processing technologies (3D printing, electrospinning), it has become a research hot spot for cartilage tissue engineering scaffold materials in recent years. The detailed mechanism of photo-crosslinkable hydrogels for bioprinting bone and cartilage tissues is shown in Figure 1 in which nanoparticles are applied as functional material carriers.

In this review, we summarize the applications of 3D bioprinting photo-crosslinked hydrogels in the repair of cartilage defects and discuss existing problems and solutions. Finally, the prospects for photo-crosslinking hydrogels in 3D bioprinting for the repair of bone and cartilage defects are outlined.

## 2. 3D Printing Photo-Crosslinked Hydrogels and Repair of Cartilage Defects

In this section, we focus on the advantages of 3D bioprinting photo-crosslinked hydrogels compared with other hydrogels and their application in the repair of cartilage defects.

### 2.1. Materials for Photo-Crosslinkable Hydrogels

The photo-crosslinked hydrogel system consists of three parts: polymerizable material, a photoinitiator, and light. The three components are inseparable and indispensable [24]. The photoinitiator is a kind of substance that can change into a high-energy state and then induce chemical changes and finally produce active intermediates with the ability to initiate polymerization after absorbing the light energy of a specific wavelength. The different types of active intermediates produced by the photoinitiators can be divided into radical photoinitiators and cationic photoinitiators. The light required for the light initiators is divided into ultraviolet light (UV) sources and visible light sources according to the emission spectrum. The commonly applied photoinitiators and light are listed in Table 1.

So far, there are many kinds of materials that have been used in the construction of photo-crosslinked hydrogels, which are generally divided into natural and synthetic materials. Natural materials, such as hyaluronic acid, alginate, and chitosan have good biocompatibility. They can interact with cells through specific surface receptors to promote cell migration, proliferation, and the production of the extracellular matrix [35,37]. Synthetic polymers mainly include polyethylene glycol, polyvinyl alcohol and its derivatives, as well as degradable block copolymers [24,28], which have excellent mechanical properties, adjustability, and availability. While providing a highly hydrated environment with the function of immune isolation, they have a high potential to trap living cells. They also can promote the diffusion of nutrients and stimulate cell migration, proliferation, and differentiation. The most commonly used method to prepare crosslinked hydrogels is based on functionalized polymers (methyl) acrylate through free radical polymerization [33,34]. Several commonly used photo-crosslinked hydrogels for cartilage repair are described below.

#### 2.1.1. Hyaluronic Acid

**Figure 2:**
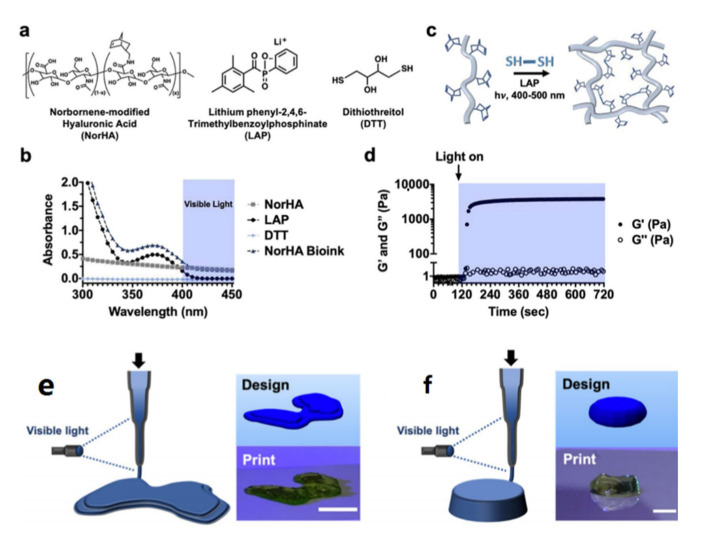
NorHA bioink composition and crosslinking. (**a**) Chemical structures of components incorporated into NorHA bioinks and their (**b**) absorption spectra, including for NorHA, LAP, DTT, and their combination, into a single bioink formulation (triangle). (**c**) Schematic of thiol-ene reaction employed to crosslink the NorHA bioink. (**d**) Representative photorheology time sweep during the photo-crosslinking of the NorHA bioink. (**e**) Schematic of in situ crosslinking method and CAD design and representative image of a printed construct for designs of a model femoral condyle and a disc (**f**). Reproduced with permission from Galarraga J. H., Biofabrication; published by IOP publishing Ltd., 2022.

HA is a natural glycosaminoglycan polymer with D-glucuronic acid and N-acetyl-D-glucosamine as disaccharide structural units. It is one of the major components of the ECM and has a high content in animal tissues such as brain tissue, synovial fluid, and the vitreous body. HA plays an important role in many biological processes such as cell proliferation, differentiation, morphogenesis, inflammation, and wound healing [38]. A large number of the carboxyl and hydroxyl groups on HA molecules also provide rich chemical sites for chemical modifications, such as the introduction of methacrylate group [39], -COOH or -NH_2_ as adhesion groups [40], the transforming growth factor-beta (TGF-β) superfamily [41] or the insulin-like growth factor-1 (IGF-1) [42,43] in drug/active factor grafting, and so on, making it an excellent platform material. The presence of vinyl groups, contributed by methacrylate in the molecular structure of methacryloylated hyaluronic acid (HAMA) gives it a light-curing ability [44]. It solidifies into gel within 10 s under visible light irradiation. Due to the portable forming method and good biocompatibility, materials based on HAMA have been applied to many biomedical fields, including in chondrocyte culture and cartilage regeneration, tumor model construction, drug-controlled release, microneedle preparation, wound dressing, biosensors, and postoperative anti-adhesion [45,46,47,48]. Galarraga et al. used a norbornene-modified hyaluronic acid (NorHA) macromer as a representative bioink and varied the printing parameters (e.g., capillary length, flow rate, light intensity) to identify printing conditions that were optimal for the ink [49], and then marrow mesenchymal stem cells (MSCs) were encapsulated in the bioink and the compound bioink was fabricated using photo irradiation for cartilage repair. Detailed information on photo-crosslinked NorHA is introduced in Figure 2. 

#### 2.1.2. Silk Fibroin

Silk fibroin (SF) is a high molecular polypeptide composed of a variety of amino acids [50]. SF molecules include a hydrophobic peptide chain (H-chain) and a hydrophilic peptide chain (L-chain). The special amino acid sequences of the H-chain and the L-chain make it possible to form a variety of protein secondary conformations. Different structures can be transformed into each other and are closely related to the solubility, degradability, chemical stability, and mechanical properties of silk fibroin materials. Various properties of silk fibroin materials can be effectively controlled by regulating the secondary structure of silk fibroin, including the preparation of high-strength and high-orientation materials [51]. SF has the characteristics of good biocompatibility, biodegradability, and high tensile strength. It has been used in various biomedical fields, including wound dressing, artificial blood vessels, cell culture, and so on. 

Methacryloylated silk fibroin (SilMA) is the methacryloylation modification of SF by glycidyl methacrylate and the introduction of double bonds on SF molecules. Due to the special spatial structure of an SF molecule, it is easy to form crystals before modification and difficult to dissolve in water. After introducing additional hydrophilic chemical groups, it can be quickly dissolved in water, which enables SilMA to be solidified into hydrogels by light. It solidifies into the gel with good biocompatibility and strong material scalability within 10 s under visible light irradiation. 

#### 2.1.3. Alginate

Alginate is a kind of polysaccharide carbohydrate extracted from kelp or Sargassum from brown algae. It is characterized by a low price, good biocompatibility, low immunogenicity, and easy access. Alginate is widely used in various biomedical fields [52]. However, there are still many deficiencies, such as poor cell adhesion, lack of osteogenic induction, and so on. However, studies have shown that polypeptide organic molecules cannot significantly enhance the adhesion of cells on the surface of hydrogels, and thus cannot effectively promote osteogenic cell differentiation [53]. Bioactive magnesium [54], strontium [55], and other divalent cations [56] were introduced into the photo-crosslinked alginate hydrogel to improve their adhesion properties through ionic crosslinking. The results show that a certain number of divalent cations can significantly improve the adhesion rate of cells on the hydrogel surface but the cell extension state is still not ideal. Tan et al. [57] introduced methacryloyloxyethyl trimethylammonium chloride and sodium methacrylate into polyethylene glycol scaffolds to prepare new materials loaded with positive and negative charges, respectively. Their results showed that cell adhesion, extension, proliferation, and differentiation on the surface of scaffolds were significantly improved. Yuan et al. [58] prepared a dual-network bovine serum albumin/sodium alginate with a hydroxyapatite nanowires composite (B-S-H) hydrogel scaffold for cartilage repair. The obtained B-S-H hydrogel scaffold exhibits ideal physical properties, such as excellent mechanical strength, a high porosity and swelling ratio, as well as excellent biological activity to promote the proliferation and differentiation of human bone marrow-derived mesenchymal stem cells (hBMSCs). Composite materials based on sodium alginate have great potential for the repair of cartilage defects.

#### 2.1.4. Chitosan

Chitosan (CS) is a natural aminopolysaccharide biomaterial with good biocompatibility and degradability, a variety of sources, a low price, and easy availability. Its molecular chain segment is rich in hydroxyl, amino, acetylamino, and glycosidic bonds [59], which makes it easy to combine with a variety of inorganic and organic molecules and it is widely used in food and medicine, as well as in environmental, biological, electrical and other fields [60]. There are many ways to modify CS to obtain photo-crosslinkable derivatives. For example, methacrylamide CS (CSMA) is synthesized by the reaction of the primary amine group of chitosan with methacrylic anhydride [61]. Qi et al. Preparation of Chitosan Graft methacrylglycine photo-crosslinking hydrogel [62]. In addition, in the presence of a biocompatible photoinitiator, chitosan [63] based on a light click mercaptan hydrogel was synthesized by photo-crosslinked maleic acid CS and thiol-terminated polyvinyl alcohol (TPVA). Zhong et al. [64] also synthesized new biodegradable hybrid hydrogels fabricated in an aqueous solution via long-wavelength UV photo-crosslinking using maleic CS and polyethylene glycol diacrylate (PEGDA) as precursors and demonstrated that the maleic CS/PEGDA hybrid hydrogel was pH-sensitive, had low cytotoxicity, and could tune the swelling, mechanical, and morphological properties of the resulting hybrid hydrogels by varying the feed ratio of maleic chitosan to PEGDA and the molecular weight of PEGDA.

In the experiment of YOO et al. [65]. A Pluronic/CS hydrogel was prepared by employing di-acrylate Pluronic and acrylate CS for thermo-responsive and photo-crosslinkable in situ gelation. They mixed acrylonitrile polysaccharides and acrylonitrile CS and transformed them into a physical gel at elevated temperatures. The gelation temperature of hydrogels gradually increased by increasing the content of CS from 0% to 15% in the initial solutions. The degradation rate of hydrogels with low photo-crosslinking time is low, and the content of chitosan in hydrogels also retards the degradation rate of the hydrogels, which is caused by highly interconnected polymer networks between acrylonitrile polysaccharides and acrylonitrile CS. Rickett et al. [66] synthesized two aryl azide CS (Az-chitosan) by conjugating 4-azidobenzoic acid with low- and high-molecular-weight CS and these solutions formed a hydrogel in less than 1 min under UV light. Lee et al. [67] prepared CS/Pluronic hydrogels by photo irradiation, which acted as injectable depot systems for gene therapy, to enhance local transgene expression at injection sites after carrying DNA in them.

#### 2.1.5. Gelatin

Gelatin is a heterogeneous mixture of polypeptides obtained by the partial hydrolysis of type I collagen [68]. Because of its appealing high biocompatibility, biodegradability, low cost, easy availability, and nontoxicity after degradation [69], gelatin is widely used in the fields of medicine and food, including as a plasma substitute in clinics and a stabilizer for protein preparations such as vaccines [70]. Unlike collagen, the antigenicity of gelatin is significantly reduced due to heating denaturation, so it is not easy to cause an immune response in the body. However, it retains the cell adhesion properties of collagen and the attachment sites of matrix metalloproteinases [71]. Therefore, the gelatin matrix can promote cell migration, proliferation, and differentiation and can trigger cell-mediated enzymatic degradation [72]. In the structure of gelatin, the carboxyl groups in aspartic acid and glutamic acid, the hydroxyl groups in serine and threonine, and the amino groups in lysine can be used for a variety of modifications and exert the photo-crosslinkable ability. Of the number of ways of modifying gelatin, methacrylate anhydride modification is the most commonly used method and through which photo-crosslinked gelatin methacryloyl hydrogels (GelMA) can be obtained [73,74,75,76].

Besides GelMA alone, there are many other materials that can be conjugated with gelatin and be made as photo-crosslinked gelatin hydrogels. For example, in the experiment of Li et al. [77], the prepared acrylate gelatin and thiolated gelatin were modified with acrylate anhydride and cysteamine, respectively. These two precursor mixtures were then crosslinked in the presence of UV irradiation and a photoinitiator. According to the results of García-Astrain et al. [78], gelatin was modified with a furan containing chromophore MFVF(5-[2-(5-methyl furylene vinylene)]furancarboxyaldehyde) and then a Schiff base formation between the primary amino groups of gelatins and the aldehyde groups of MFVF was conducted. Interestingly, this photosensitive crosslinkable hydrogel can be prepared by UV-light irradiation in the absence of initiators. In other research by Greene et al. [79], a highly tunable gelatin-based hydrogel was prepared using orthogonal thiol-norbornene photochemistry. AnilKumar et al. used furfuryl-gelatin as a visible-light crosslinkable hydrogel and rose bengal or riboflavin as a visible-light photoinitiator in the process of hydrogel formation [80]. 

Levett et al. [81] synthetized a photo-crosslinking GelMA/HAMA composite hydrogel by combining GelMA and HAMA and then human chondrocytes were encapsulated in it for 3D culture. Suo et al. [82] prepared a photo-crosslinked GelMA/AGA gelatin when gelatin was loaded with acrylamide-based glucose (AGA) and the results showed that GelMA/AGA was more stable because the swelling rate, gelatin solubility, and hydrogel degradation rates were lower. Visser et al. [83] dissolved horse cartilage, meniscus, and tendon tissue after a decellular treatment to modify methacrylic anhydride and form a new composite hydrogel with GelMA and used it to culture horse chondrocytes and bone marrow mesenchymal stem cells (BMSCs). Gao et al. [84] synthesized the photo-crosslinked methyl acrylic polyethylene glycol (PEG)/GelMA hydrogel and proved that the compression modulus of PEG/GelMA was 10 times higher than GelMA alone. In a study by Boere et al. [85], a novel composite hydrogel containing Polymethylacryloyl poly (hydroxymethylhexyl ester-ε-Caprolactone)-Poly(ε-Caprolactone) (pMHMGCL/PCL) and GelMA was prepared by covalent crosslinking using UV irradiation. Bartnikowski et al. [86] combined alginate/hydroxyapatite (HAP), GelMA, and GelMA/HAMA to prepare two novel composite photo-crosslinked hydrogels named GelMA-alginate/HAP or GelMA/HAMA-alginate/HAP hydrogels and confirmed that the composite hydrogels increased mechanical strength without affecting the proliferation and synthesis of chondrocytes.

#### 2.1.6. Synthetic Materials

As the representatives of synthetic materials, including poly(D, L-lactide) (PDLLA), poly(Ɛ-caprolactone) (PCL), polyvinyl alcohol (PVA), PEG, poly(trimethylene carbonate) (PTMC), poly(ethylene carbonate) (PEC), and block copolymers containing PEG, poly(propylene glycol) (PPG) or poly(tetramethylene glycol) (PTMG) and poly(glycolide) (PGA), and PDLLA or PCL segments, PEG and PVA are the most commonly applied materials to be used to prepare hydrogels. Although the bioactivity of synthetic materials is insufficient compared with natural materials, their chemical and mechanical properties are repeatable, consistent, and adjustable. Therefore, synthetic materials are mostly combined with other natural materials to be synthesized as compound materials [87]. 

PEG, also called polyethylene oxide or polyoxyethylene, is a high molecular (600 Da~100 kDa) polymer with the chemical formula HO (CH_2_CH_2_O) nH that does not irritate, has a slightly bitter taste, is hydrophilic, and has good compatibility with many organic components. It has excellent lubricity, moisture retention, dispersion, and adhesion and is widely used in pharmacy, food processing, clinical treatments, and so on. PEG-based hydrogels are flexible and widely used in wound dressing, tissue scaffolds, cell or drug delivery, and medical implants in the biomedical field, and they rarely cause inflammation [88]. The main advantages of PEG application in tissue engineering include its adjustable structural and mechanical properties, biocompatibility, hydrophilicity, low cytotoxicity, and non-immunogenicity [89]. Because PEG is nondegradable and has inadequate adhesion sites for cells, it is generally compounded with other materials to develop bioinks. Bal et al. [90] used several types of peptides to mortify PEG hydrogels and found that the morphology of MSCs remained spherical in PEG hydrogels and no significant changes were observed until day 5.

PVA is also a hydrophilic and linear synthetic ethanol homopolymer. A large number of side hydroxyl groups provide attachment sites and possible modifications for biomolecules. Hydrogels from PVA or other derivatives of PVA have been widely used due to their chemical modulation. According to some research [71], pure PVA hydrogels were unable to provide long-term cell growth because from days 1 to 14, the cell viability of MSCs decreased from 87% to 71%. After binding to GelMA, the cell viability reached 92% on day 14. The composition, crosslinking mechanism, advantages, and disadvantages of each material are shown in Table 2.

### 2.2. Advantages of Photo-Crosslinked Hydrogels

The formation process of hydrogels is mainly mediated by the physical or chemical crosslinking of polymers. According to the formation mechanism, hydrogels are divided into a physical crosslinking hydrogel and a chemical crosslinking hydrogel. Physical crosslinking hydrogels, prepared by a traditional method, are formed by non-covalent bonds such as hydrogen bonds and Coulomb or Van der Waals forces. The crosslinking effect is relatively weak and not sensitive to external environmental changes, so it has a certain reversibility. Chemical crosslinking refers to the interaction between polymers under the stimulation of force, light, heat, high-energy radiation, ultrasound, and so on. The crosslinking point of the chemical bond is formed through a covalent bond and then the three-dimensional network structure is formed through the use of the crosslinking agent. The degree of crosslinking is strong with long-term stability and its own physical structure or chemical properties change significantly. 

**Figure 3:**
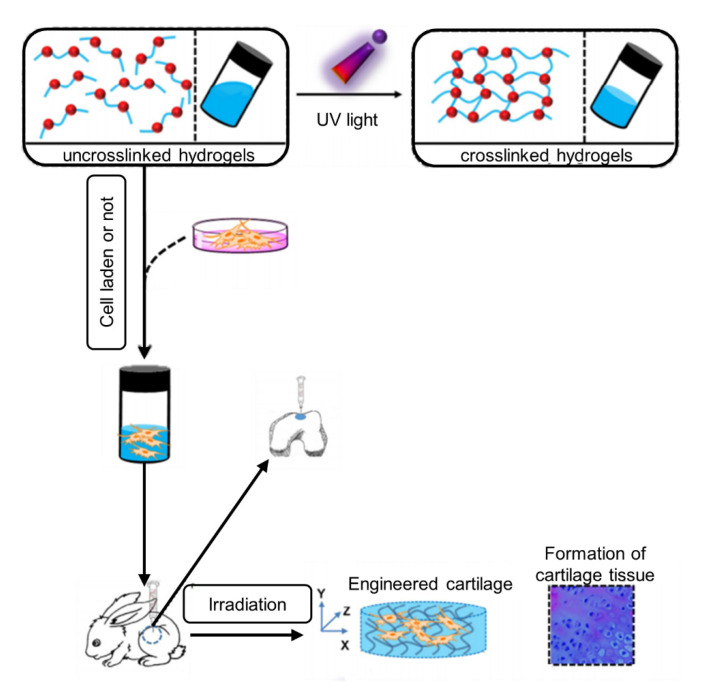
Schematic diagram of preparation and utilization of photo-crosslinked hydrogel as a 3D scaffold for engineering artificial cartilage in situ [27], reproduced with permission from Chao Qi, Biomaterials; published by Elsevier, 2018.

Among all chemical crosslinking types, whether through triggers, such as heating, changing the pH, and introducing specific ions, or through spontaneous processes, such as sulfhydryl maleimide, aldehyde, amino, and amino epoxy two-component crosslinking, photo-crosslinked hydrogels have the advantages of non-physical contact and precise control in time and space, thus giving the hydrogel precise processing and real-time in situ crosslinking characteristics. In addition, because of their low toxicity and high crosslinking efficiency, the photo-crosslinked hydrogels can be applied in different shapes and sizes according to their needs and can be cured in situ, which is the most outstanding advantage of photo-crosslinked hydrogels (Figure 3). They have the advantage of controllable and convenient clinical application and have become one of the hot research directions for hydrogel materials [91]. 

Moreover, the crosslinking density and physicochemical properties of photo-crosslinkable hydrogel can be precisely controlled by adjusting the intensity of light and exposure time to promote cell proliferation and differentiation [92,93]. In the research of Duchi et al. [94], the hydrogel was crosslinked in only 10 s by UV light and the viability of the cell encapsulated in it remained above 90% after 7 days of printing. With the application of UV light or visible light, the photo-crosslinking hydrogels undergo rapid formation immediately after printing [95], which is the other advantage of photo-crosslinked hydrogels compared with other hydrogels. Furthermore, by changing the light intensity and exposure time, the performance of hydrogels including the crosslinking density and matrix stiffness can be exceptionally controlled.

### 2.3. Applications of 3D Bioprinted Photo-Crosslinkable Hydrogels for Osteochondral Regeneration

Cartilage in the joints is a smooth and elastic tissue with a poor self-repair ability. Usually, a super-physiological shock load and osteoarthritis result in cartilage defects. Cells in cartilage tissue consist of only a single species and have a low number and low proliferative activity of chondrocytes, resulting in the cartilage regeneration being limited by its low cellularity and avascular nature [85]. Except for osteoarthritis, most cartilage defects are accompanied by subchondral bone defects. However, it is well known that cartilage defects involving cartilage and subchondral bone are difficult to repair due to differences in physiological structure and bioactive properties. This is because the cartilage tissue has no blood vessels and nerves but the subchondral bone tissue is rich in blood and nerves. Damage to one tissue results in impaired function of the other due to the tight bonding between the subchondral bone and the cartilage tissue. In addition, the structure and function of cartilage and subchondral bone are also different [96]. At present, arthroscopic chondrocyte transplantation and autologous osteochondral transplantation are common methods for the treatment of patients with grade III-IV cartilage defects according to the International Cartilage Repair Association cartilage damage classification system (ICRS) [97]. Cartilage tissue engineering provides a new choice for articular cartilage regeneration in osteoarthritis or traumatic injury [98,99].

**Figure 4:**
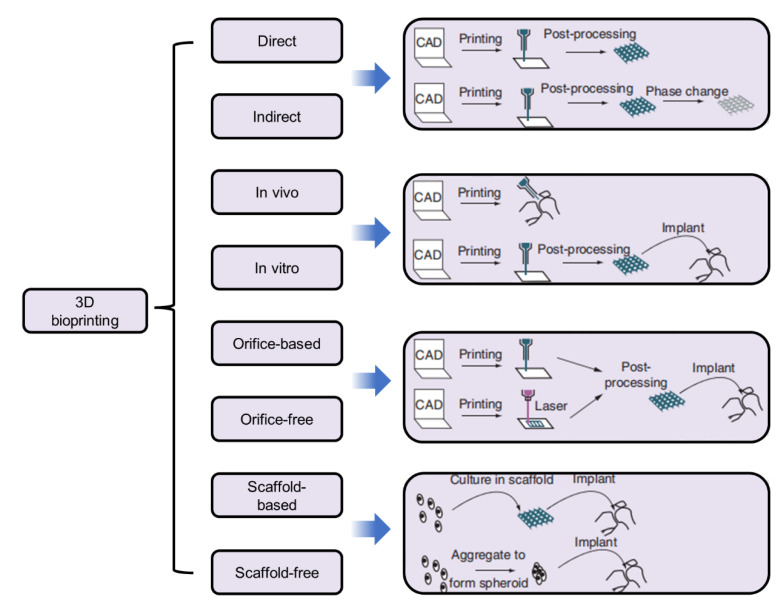
Classifications of 3D bioprinting according to various characteristics and their design and application [101]. Reproduced with permission from Ji Xiongfa, Regenerative Medicine; published by Future Medicine Ltd., 2018.

As a novel 3D technology, bioprinting has received increasing attention worldwide and is widely applied for broad-spectrum applications in regenerative medicines, tissue engineering and transplantation, the pharmaceutical field [100], and the classifications of 3D bioprinting according to various characteristics. Their design and their applications in different fields are shown in Figure 4. 

Based on their precise deposition and administrative advantages, biomaterials, viable cells, drugs, and growth factors are concurrently deposited within a computer-aided layer-by-layer stacking pattern to mimic natural constructs such as skin, bone, cartilage, lung, liver, and cardiac tissue [102]. As the most important element of 3D bioprinting, the characteristics of bioink are particularly important. In many bioink materials, hydrogels are considered ideal bioinks for bioprinting due to their adjustable physical-mechanical properties, high water content, and advanced biodegradability, especially hydrogels with 3D crosslinked networks, which makes them an kiessential component of bioink, as they can directly contact cells, keep cell activity, and support their adhesion, growth, and proliferation [103]. 

#### 2.3.1. Cartilage-like Tissue Hydrogels

As mentioned above, HA, gelatin, alginate, CS, and SF are the main hydrogel materials for osteochondral tissue engineering. These materials can be used alone or with other substances to synthesize photo-crosslinked hydrogels. Visscher, et al. [104] applied a kind of photo-crosslinkable cartilage-derived ECM (cdECM) bioink for cartilage tissue engineering. This cdECM was subsequently processed into a photo-crosslinkable hydrogel using methacrylation cdECM (cdECMMA) and mixed with chondrocytes to create a printable bioink. They found that auricular chondrocytes in the printed cdECMMA hydrogel constructs maintained their viability and proliferation capacity and eventually produced cartilage ECM components, including collagen and glycosaminoglycans (GAGs). Qi et al. [27] synthetized a photo-crosslinkable, injectable sericin hydrogel as a 3D biomimetic ECM for the minimally invasive repair of cartilage defects. In their study, sericin was functionalized to be sericin methacryloyl (SerMA), which formed an in situ hydrogel upon UV light irradiation via photo-crosslinking. They found that SerMA hydrogels were adhesive to chondrocytes and promoted the proliferation of attached chondrocytes even in nutrition-lacking conditions and that notably, the in vivo implantation of chondrocyte-laden SerMA hydrogels effectively formed artificial cartilage after 8 weeks, which molecularly resembled native cartilage. Moor, et al. [105] applied photo-crosslinked GelMA as the hydrogel scaffold to encapsulate and culture human BMSC. They found that the encapsulation in GelMA resulted in further chondrogenic maturation observed by increased production of GAG and a reduction in collagen I. After 3D bioprinting, the spheroids of human BMSCs remained and the cartilage phenotype was observed. They concluded that human BMSCs were able to differentiate into cartilage and display a geometry compatible with 3D bioprinting. Moreover, for the bioprinting of these spheroids, GelMA was a promising material as it exhibits favorable properties in terms of printability, supports the viability and chondrogenic phenotype of human BMSC microtissues, and the lower hydrogel stiffness enhanced further chondrogenic maturation after 3D bioprinting.

#### 2.3.2. Stem Cell Encapsulation Hydrogels

Damme et al. [106,107] developed a norbornene-thiol modified gelatin hydrogel to be used for cell encapsulation purposes [108,109]. The cell viability of human adipose-derived stem cells(hASCs) encapsulated in all the hydrogels at every time point exceeded 85% according to the Calcein Acetoxymethyl Ester (Ca-AM)/Pyridine iodide (PI) staining analysis. Although their research did not induce hASCs into chondrocytes, the hydrogel has superior load cell capabilities. If we add cytokines into the hydrogel that can induce the differentiation of hASCs into chondrocytes, then the hydrogel can immediately be applied to the repair of osteochondral defects. Levato et al. [110] synthetized a novel set of photo-responsive bioresins derived from ichthyic-origin gelatin and after encapsulating BMSCs in them, 3D printing was exerted via digital light processing (DLP). The results of the 3D culture showed that the hydrogel had excellent cytocompatibility. At every detection time, the proportion of live cells in all the hydrogels was greater than 75%, indicating that the hydrogel had no obvious cytotoxicity. Moreover, the encapsulated BMSCs were able to retain their multi-lineage differentiation capabilities when exposed to media derived from chondrogenic, hypertrophic, and osteogenic differentiation protocols. For example, positive collagen II staining can be tested when cultured in a chondrogenic medium, and the ECM deposition appeared diffused throughout the whole hydrogel matrix. Custodio et al. [111] reported an approach to synthesizing photo-crosslinkable laminarin hydrogels by chemical modification with acrylate groups. Similar to Damme et al., in their study, they encapsulated hASCs in this hydrogel and they found a uniform distribution of >90% of viable cells (in green) encapsulated in the photo-crosslinked hydrogels according to live dead staining and interestingly, cell viability with various concentrations of laminarin or degrees of methacrylation remained constant, which confirmed the biocompatibility of methacrylate laminarin for cell encapsulation. 

#### 2.3.3. Cartilage Tissue Cell Encapsulation Hydrogels

Gao et al. [84] developed a unique inkjet bioprinting approach to creating mechanically strong bone and cartilage tissue constructs using PEG dimethacrylate, gelatin methacrylate, and human MSCs (hMSCs). The findings showed that inkjet-bioprinted-hMSCs in simultaneously photo-crosslinked PEG-GelMA hydrogel scaffolds demonstrated an improvement in mechanical properties and osteogenic and chondrogenic differentiation, suggesting their promising potential for usage in bone and cartilage tissue engineering. Kim et al. [112] reviewed the application of SF and they also reported that human chondrocytes were encapsulated in methacrylated SF solution (Sil-MA) hydrogels and cultivated in vitro. This cell-laden Sil-MA hydrogel showed great cartilage tissue formation. Galarraga et al. [49] applied NorHA macromer as a representative bioink and developed an approach termed in situ crosslinking that permits the printing of non-viscous, photo-crosslinkable bioinks via the direct curing of the bioink with light through a photopermeable capillary prior to deposition and encapsulated human MSCs in them. Over 56 days of culture in chondrogenic media, the printed constructs increased in compressive moduli, biochemical content (i.e., sulfated glycosaminoglycans, collagen), and histological staining of the matrix associated with cartilage tissue. Chen et al. [113,114] mixed water-based thermoplastic polyurethanes and water-based light-cured polyurethanes and developed a light-curing waterborne polyurethane hydrogel for the construction of 3D-printed cytocompatible cartilage scaffolds. The results showed a good cell adhesion amount and viability and the hydrogel can be used for cartilage tissue engineering. 

Setayeshmehr et al. [115] applied two methods to develop novel, cell-compatible dual-component biomaterial inks and bioinks based on PVA and solubilized decellularized cartilage matrix (SDCM) hydrogels that can be utilized for cartilage bioprinting. They. The first method was that PVA was modified with amine groups (PVA-A) and mixed with SDCM(PVA-A/SDCM). The other method was that the PVA was functionalized with cis-5-norbornene-endo-2,3-dicarboxylic anhydride (PVA-Nb) to allow ultrafast light-curing thiol-ene crosslinking and then mixed with SDCM(PVA-Nb/SDCM), and the teratocarcinoma-derived chondrogenic cell line (ATDC5) was encapsulated in these two hydrogels. From their results, all hydrogel formulations showed over 70% cell viability at all time points measured and a round morphology was observed seven days after the encapsulation of the ATDC5 cells according to alcian blue and a fast red stain. Cho et al. [116] developed a new photo-crosslinkable glycol chitosan thermogel for biomedical applications named methacrylated hexanoyl glycol CS (M-HGC), which was synthesized by a series of chemical modifications, using N-hexanoylation and N-methacrylation, of glycol CS (GC). These thermally induced hydrogels could be chemically crosslinked by UV-triggered photo-crosslinking. According to the feed molar ratio of glycidyl methacrylate (GM) to the glucosamine residue of GC, the M-HGC hydrogels were divided into M5-HGC, M8-HGC, and M20-HGC. After the hydrogels were prepared, rabbit chondrocyte cells were encapsulated in the M-HGC hydrogels by suspending the cells in the M-HGC solution and then photo-crosslinked with UV irradiation for 15 min. After the cells had been cultured for 1, 3, and 7 days in the M-HGC hydrogels, GC, HGC, M5-HGC, and M8-HGC showed low cytotoxicity at various concentrations for chondrocytes. According to the MTT assay, the cytotoxicity of M5-HGC, as measured with the methyl thiazolyl tetrazolium (MTT) assay, was not observed even at a high concentration (1 mg/mL) over the culture time and round chondrocytes were uniformly distributed in the M5-HGC hydrogel. Most of the encapsulated chondrocytes were shown to survive in the M5-HGC hydrogel after the photo-crosslinking process (day 1). In addition, a higher number of live cells were observed at 3 and 7 days of culture than after 1 day of culture according to the live/dead assay. These results may be because the M5-HGC hydrogel did not exert any significant toxicity on the encapsulated cells.

## 3. Problems with 3D Bioprinting Photo-Crosslinked Hydrogels 

Although the photo-crosslinked hydrogels have numerous advantages, compared with other types of hydrogels there are still some problems in the application of photo-crosslinked hydrogels for 3D bioprinting. In this section, we focus on discussing 3D bioprinting-related problems and the disadvantages of photo-crosslinked hydrogels for the repair of osteochondral defects. More and more researchers have pointed out that it is doubtful that encapsulated cells can effectively repair osteochondral damage in hydrogels. Therefore, loading cells in hydrogels is a controversial issue in the field of the repair of osteochondral defects.

### 3.1. Cell Viability and Loaded Cells

In osteochondral tissue engineering, the most commonly used cells are MSCs. MSCs have unlimited proliferation and differentiation potential and have become the best choice for 3D bioprinting. However, compared with mature somatic cells, MSCs respond more strongly to harsh environments but are more unlikely to survive [117]. For animal cells to survive in vitro they have extremely high requirements for the surrounding environment. Firstly, they must be sterile and non-toxic. Secondly, they must have sufficient nutrients, including protein, sugar, fat, vitamins, etc., as well as an appropriate temperature, pH, osmotic pressure, and a certain concentration of O_2_ and CO_2_. In addition, under the special conditions of 3D bioprinting, cell viability is also affected by many other factors, such as cell-loading technologies and the construction process. 

#### 3.1.1. Bioink and Cell Viability

The viability of the cells encapsulated in the hydrogels is affected by their type, concentration, viscosity, rigidity, type of crosslinking agent, and the crosslinking degree of the hydrogels. Compared with synthetic hydrogels, natural hydrogels have better biocompatibility and higher cell viability. KOCH et al. [118] studied the effects of alginate, collagen, fibrin, HA, and other hydrogels on cell viability, and found that HA-based hydrogel had the best cell survival rate. Zhang et al. [119] and Park et al. [120] studied the effects of the alginate concentration and composition on cell viability. Their results showed that a low concentration was more conducive to maintaining cell viability and morphology and that when the ratio of the high to low molecular weight of alginate was 2:1, it was most conducive to cell proliferation and differentiation. Hydrogels have the property of being porous with a low concentration, low viscosity, low rigidity, and low crosslinking, which is more conducive to the diffusion of oxygen, nutrients, metabolic wastes, and cell migration. At the same time, the distribution of cells in the hydrogels is also affected by the viscosity of the hydrogel solution. In low-viscosity hydrogels, cells have a tendency to deposition and poor dispersion, which is not conducive to cell differentiation and proliferation or the production of the cartilage matrix. Ultimately, the agglomeration of cells affects the repair effects of articular cartilage. So, one of the main challenges in bioprinting is cell sedimentation to ensure a homogenous distribution of cells within a printed hydrogel [121].

#### 3.1.2. 3D Bioprinting Methods, Parameters, and Cell Viability

No matter what kind of 3D printing method is used, the printer will induce a certain shear force on the bioink in the process. The application of different bioinks results in different shear stresses. At the same time, the shear stress is also related to the printing rate, resulting in varying degrees of damage to the cell viability. Although the encapsulating hydrogel has a protective effect on the cells, the influence of the shear stress or mechanical cytotoxicity coming from the extrusion or the bioprinting process is still unavoidable [122]. The massive cell death upon delivery is caused by shear stress from the needle, poor engraftment of delivered cells, and, as a consequence, limited ability to differentiate into a chondrogenic phenotype [123,124]. High shear stress can cause cell deformation and even destroy the cell membrane, resulting in low cell viability [125]. Different printing methods produce different shear stresses and cell viabilities. Due to the higher shear stress, the cell viability of extrusion 3D bioprinting is only 40–80%, whereas the cell viability of inkjet and laser-assisted 3D bioprinting is higher than 85% and 95%, respectively [126].

For the same printing method, the printing parameters also have significant effects on cell viability. The diameter, shape, spraying speed, distribution pressure, and exposure time of the extrusion needle can influence the shear stress and affect cell viability. In addition, the hole and pore sizes, shape, and porosity of the printing structure will also affect the survival and biological behavior of the cells. For example, the small-diameter extrusion needle can increase the accuracy of the print structure, but it will increase the shear stress and extrusion stress on the cell, which will ultimately affect the cell viability of the cells encapsulated in it [127]. In the research of Shen et al., results showed that the shear stresses produced by extrusion needles with different shapes were also different. Compared with a cylindrical extrusion needle, the conical extrusion needle produces less shear stress and less damage to cells [128]. In addition, low injection speed, low distribution pressure, and low exposure time were more conducive to cell survival [125]. Moreover, hydrogels with high porosity and large pore sizes were more conducive to material exchange and cell proliferation.

### 3.2. Cytotoxicity of Photo-Crosslinked Hydrogels

#### 3.2.1. Free Radical Toxicity

During 3D bioprinting, light exposure induces the photoinitiator to generate free radicals, which may impair cells [129,130]. Studies have shown that photoinitiators in photo-crosslinked hydrogel systems can cause damage to cell membranes, nucleic acids, and proteins, which can cause damage to cells and even lead to cell death [131]. In addition, high-energy free radicals can induce organic compounds to transform into reactive oxides, resulting in oxidative damage to the cells or other active substances in the hydrogel [132]. Elisseeff et al. found that different types of cells have different responses to the same concentration of photoinitiators, and the faster the proliferation rate, the more sensitive the free radicals generated by the photoinitiators [133]. 

#### 3.2.2. Phototoxicity

The photo-crosslinking process commonly employs a UV light source, which is itself a potential source of cytotoxicity due to UV-induced apoptosis and, most importantly, genotoxicity when DNA-damaged cells are not eliminated. However, some scientists mentioned that without the photoinitiators, cellular viability has barely been affected by the illumination, whether the light wavelength is UV or Blu-ray. Therefore, this demonstrates another advantage for the application of photo-crosslinked hydrogels. Long-wave UV (315–400 nm) is widely accepted as a mutagen owing to its ability to induce cellular DNA damage, and shortwave UV (B, 280–315 nm) irradiation can lead to DNA base lesions such as cyclobutane pyrimidine dimers (CPDs) and pyrimidine 6-4 pyrimidone photo-products [134].

Recently, the cytotoxicity of UV radiation was tested in human MSCs and the results showed that prolonged exposure to high-intensity UV radiation (370 ± 5 nm; 788 kJ/m^2^) in the absence of any photoinitiators, resulted in a significant reduction in cell viability of up to 50% compared with cells exposed to visible light only [135]. Therefore, the longer the UV exposure lasts, the more serious the problem of cytotoxicity.

#### 3.2.3. Cytotoxicity of Photoinitiators

The photoinitiator itself has intrinsic toxic effects although this varies between photoinitiators and can be minimized by selecting the lowest practicable concentration [136]. In the absence of photoinitiators, cell viability is hardly affected by light, whether the light wavelength is ultraviolet or blue; however, under the action of irgacure 2959 and ultraviolet radiation, the cultured cells were damaged to varying degrees. It was found that among the commonly used photoinitiators, irgacure 651 had the greatest cytotoxicity, whereas irgacure 2959 with a relatively polar structure had the least cytotoxicity [112]. In addition, if the light-curing reaction is not complete, the toxicity of the photoinitiator will increase, and the higher the number of photoinitiators, the less conducive they are to cell growth [113].

**Figure 5:**
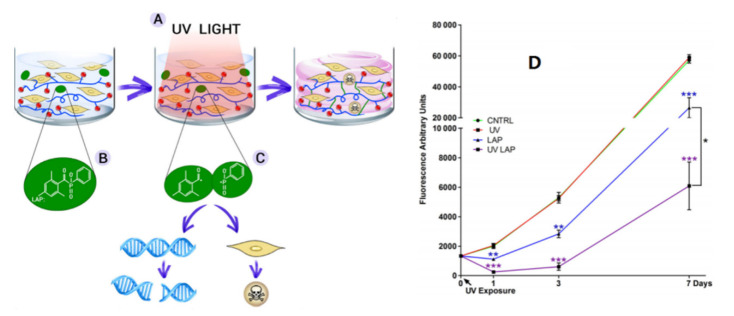
(**A**–**C**) Schematic representation of the photo-crosslinking process of a hydrogel laden with cells and cell cytotoxicity induced by the photoinitiator LAP and (**D**) UV light irradiation. * is *p* ≤ 0.05; ** is *p* ≤ 0.01; *** is *p* ≤ 0.001 [94,122].

As an example of intrinsic toxicity, the lithium phenyl-2,4,6-trimethylbenzoylphosphinate (LAP) photoinitiator molecule has been studied in relation to cellular survival [94]. Cytotoxicity was induced by LAP and UV light irradiation at 365 nm with an irradiance of 700 mW/cm^2^ for 10 s. hADSCs were cultured in a 2D medium and assayed for 7 days in culture with a metabolic test to measure the cytotoxicity induced by cell exposure to UV light alone (UV), LAP on its own (LAP), and UV exposure to LAP (UV LAP) compared with untreated cells (CNTRL). The survival of cells was highly affected by exposure to UV light at 365 nm, with an irradiance of 700 mW/cm^2^ for 10 s in combination with LAP, as well as to LAP alone, but not to UV light itself or untreated. The details are shown in Figure 5.

## 4. Solutions and Future Horizons

This review summarizes the literature related to the applications of photo-crosslinkable hydrogel-based 3D bioprinting in the repair of osteochondral defects. As mentioned in our review, numerous studies have reported that photo-crosslinkable hydrogel-based 3D bioprinting has shown good results in cell differentiation or proliferation and repairing osteochondral defects. However, it should be noted that significant challenges remain for this treatment strategy. For example, the cytotoxicity of photo-crosslinkable hydrogels to cells and the mechanical cytotoxicity of printer-induced shear force need to be investigated. 

How can these problems be resolved? In our opinion, more in-depth research on the following topics should be performed. Table 3 summarizes the current status, existing problems, and solutions of 3D-printed photo-crosslinked hydrogels.

i. Developing and exploring more biocompatible photoinitiators for visible light to minimize the damage to cells by the photoinitiators. Small doses of photoinitiators promote the crosslinking of hydrogels at very low concentrations without affecting the rate of the crosslinking reaction and the mechanical strength of the hydrogels. In addition, developing more hydrogels without external photoinitiators such as hydrogel-based photoinitiator systems that contain crosslinkable polymers to crosslink and form hydrogels. Atom transfer radical polymerization (ATRP) is a controllable radical synthesis technology catalyzed by transition metal complexes. Vinyl monomers are initiated by the initiator R-X and polymerized to form macromolecules in a reversible oxidation-reduction process [137]. Compared with traditional radical polymerization, ATRP can improve the homogeneity of hydrogels, and different structures and properties of hydrogels can be obtained by using different initiators.
ii. Adding antioxidants, such as N-acetylcysteine [138] and graphene oxide [139], and bioactive factors, such as growth factors [140,141] and polypeptides [142]. Imparting functionality to materials without compromising the performance of bio-inks.
iii. Optimizing printing methods and parameters [143,144,145], such as microencapsulation and nanoencapsulation that can encapsulate the cells in a protective shell with good biocompatibility and isolate them from the surrounding environment to reduce the stimulation of the external environment to the cells. Optimizing the diameter of the printing needle, jet speed, pore diameter, shape, and porosity of the structure to optimize the printing structure [146].
iv. Combining different gelation methods of hydrogel printing strategies to optimize the performance of composites in the process. Several hydrogels with different gelation methods were combined to optimize the performance of hydrogels. For example, the thermal crosslinking material is combined with the photo-crosslinking material to form a hydrogel composite for rapid crosslinking into a hydrogel [147,148,149]. The dual-responsive hydrogel constructs demonstrated higher resolution and shape fidelity as well as better cell viability and proliferation than the thermal responsive control.

## Figures and Tables

**Table 1:** Photoinitiator types widely used for hydrogel photo-crosslinking.

Name	Abbreviation	Light	Ref
1-[4-(2-hydroxyethoxy) phenyl]-2-hydroxyl-2-methyl-1-acetone	Irgacure 2959	UV	[25,26,27,28]
Lithium phenyl-2,4,6-trimethylbenzoyl phosphinate	LAP	UV visible light	[29,30,31,32]
2, 4, 5, 7-tetrabromofluorescein disodium salts	Eosin Y	visible light	[33,34]
2-Hydroxy-2-Methylphenylacetone	Irgacure 1173	UV	[35,36]
lactochrome	riboflavin	visible light	[37]

**Table 2:** Materials for photo-crosslinkable hydrogels.

Samples	Composition	Crosslinking Mechanism	Advantages	Disadvantages
HA	D-glucuronic acid and N-acetyl-D-glucosamine as disaccharide structural units	Cured by vinyl polymerization with the introduction of methacrylates	Abundant active sites, Machinability, Adapt to multiple printing methods	Complex modification process
SF	A variety of amino acids	Dehydration condensation of amino acids	Spatial structural controllability, High orientation, High tensile strength	Variability affected by storage conditions
Alginate	Polysaccharide carbohydrate	Introduction of cations and induction of crosslinking	Good biocompatibility, Low immunogenicity, Easy access	Poor cell adhesion, Lack of osteogenic induction
Gelatin	Heterogeneous mixture	Methacrylic acid modification induced photo-crosslinking	Non-toxicity after degradation, Promote cell migration, Proliferation and differentiation, Trigger cell-mediated enzymatic degradation	Susceptible to bacterial contamination
Synthetic materials	Polymer monomer	Polymerization of monomers	Adjustable performance, Repeatability, Suitable for production	Poor biocompatibility

**Table 3:** Prospects for photocurable hydrogel researches.

Research Status	Existing Problems	Optimization
The requirement of photoinitiator	Destruction of UV light for encapsulated cells, Biotoxicity of photoinitiators	Develop visible light photo-crosslinking method, Search for low-toxicity photoinitiators
Material selection for application environment	Insufficient functionality of materials	Physical mixing or chemical modification imparts multifunctional properties on materials
Hydrogels encapsulate cells directly	Direct exposure to the external environment is destructive to cells	Build cell protective shells to keep cells alive
Photocurable hydrogel strategies are limiting	Printing method is limited	Optimize the printing process, Combine various cross-linking processes

## Data Availability

Not applicable.

## Abbreviations

AC	articular cartilage
ECM	extracellular matrix
CTE	cartilage tissue engineering
HA	hyaluronic acid
3D	three-dimensional
2D	two-dimensional
HAMA	methacryloylated hyaluronic acid
NorHA	norbornene-modified hyaluronic acid
MSCs	marrow mesenchymal stem cells
SF	silk fibroin
SilMA	methacryloylated silk fibroin
CS	chitosan
CSMA	methacrylamide chitosan
TPVA	thiol terminated polyvinyl alcohol
PEGDA	polyethylene glycol diacrylate
GelMA	gelatin methacryloyl hydrogels
BMSCs	Bone marrow mesenchymal stem cells
PEG	polyethylene glycol
pMHMGCL/PCL	Polymethylacryloyl poly (hydroxymethylhexyl ester -ε- Caprolactone)-Poly(ε- Caprolactone)
HAP	hydroxyapatite
PVA	polyvinyl alcohol
ICRS	International Cartilage Repair Association cartilage damage classification system
cdECM	cartilage-derived ECM
cdECMMA	methacrylation cdECM
GAGs	glycosaminoglycans
SerMA	sericin methacryloyl
hASCs	human adipose-derived stem cells
Ca-AM	Calcein Acetoxymethyl Ester
PI	Pyridine iodide
DLP	digital light processing
hMSCs	human MSCs
Sil-MA	methacrylated SF solutions
SDCM	solubilized decellularized cartilage matrix
PVA-A	PVA/amine
PVA-Nb	PVA/cis-5-norbornene-endo-2,3-dicarboxylic anhydride
HGC	methacrylated hexanoyl glycol CS
GC	glycol CS
GM	glycidyl methacrylate
CPDs	cyclobutane pyrimidine dimers
LAP	lithium phenyl-2,4,6-trimethylbenzoylphosphinate
